# Differential Associations of Lipopolysaccharide, Soluble NOX2-Derived Peptide, and Hydrogen Peroxide with Adiposity and Muscularity in Adults on Maintenance Hemodialysis

**DOI:** 10.3390/antiox15091156

**Published:** 2026-09-11

**Authors:** Giovanni Imbimbo, Federica Foti, Thomas Ammann, Maria Grazia Chiappini, Vittoria Cammisotto, Valentina Castellani, Pasquale Pignatelli, Alessio Molfino

**Affiliations:** 1 Department of Translational and Precision Medicine, Sapienza University of Rome, 00185 Rome, Italy; giovanni.imbimbo@uniroma1.it (G.I.); federica.foti@fbf-isola.it (F.F.); 2 Hemodialysis Unit, Fatebenefratelli Hospital, Isola Tiberina, 00185 Rome, Italy; thomas.ammann@fastwebnet.it (T.A.); mariagrazia.chiappini.fw@fbf-isola.it (M.G.C.); 3 Department of Medical and Cardiovascular Sciences, Sapienza University of Rome, 00185 Rome, Italy; vittoria.cammisotto@uniroma1.it (V.C.); pasquale.pignatelli@uniroma1.it (P.P.); 4 Istituto Pasteur Italia—Fondazione Cenci Bolognetti, Istituto Pasteur Italia, 00185 Rome, Italy; valentina.castellani@uniroma1.it; 5 Department of Interdisciplinary Studies on WellBeing, Health and Environmental Sustainability (BeSSA) Sapienza University of Rome, 02100 Rome, Italy

**Keywords:** hemodialysis, endotoxemia, NOX2, body composition, normalized protein catabolic rate, oxidative stress

## Abstract

**Background:** Endotoxemia and oxidative stress may contribute to adverse body-composition changes in patients receiving maintenance hemodialysis, but their relationships with adiposity and muscularity remain incompletely defined. We investigated whether the concentrations of circulating lipopolysaccharide (LPS), soluble NOX2-derived peptide (sNox2-dp), and hydrogen peroxide (H_2_O_2_) showed differential associations with bioimpedance-derived body-composition compartments and explored their relationships with normalized protein catabolic rate (nPCR), a surrogate of protein intake. **Methods:** In this observational, cross-sectional study, adult patients receiving maintenance hemodialysis were enrolled at a single center. Serum LPS and sNox2-dp and H_2_O_2_ concentrations were measured before dialysis. Body composition was assessed by bioimpedance analysis; fat mass (FM) was used as an index of adiposity, whereas intracellular water indexed to height squared (ICW/h^2^) was used as a proxy of muscularity. Associations were examined using Spearman correlation and multivariable linear regression adjusted for age, sex, and nPCR. **Results:** A total of 58 participants were included with a median age of 73 years; 64% were male and mean body mass index was 24.6 ± 4.2 kg/m^2^. LPS correlated with sNox2-dp (rho = 0.420, *p* = 0.001), whereas sNox2-dp correlated with H_2_O_2_ (rho = 0.352, *p* = 0.007); the LPS–H_2_O_2_ association was borderline (rho = 0.259, *p* = 0.050). sNox2-dp correlated positively with fat-free mass, total body water, ICW, and ICW/h^2^, whereas LPS correlated with FM (rho = 0.305, *p* = 0.023). Participants with nPCR > 0.89 g/kg/day had higher LPS concentrations than those with lower nPCR (*p* = 0.015), and nPCR correlated with ICW/h^2^ (rho = 0.36, *p* = 0.007). In multivariable analysis, LPS remained independently associated with FM (β = 0.210, *p* = 0.046). nPCR was positively associated with ICW/h^2^ at the threshold of statistical significance (β = 1.393, *p* = 0.050), whereas sNox2-dp showed a nonsignificant positive trend (*p* = 0.060). **Conclusions:** In maintenance hemodialysis, endotoxemia and NOX2 activation showed differential associations with body composition: LPS with adiposity and sNox2-dp with muscularity. Higher nPCR was associated with both greater muscularity and higher LPS concentrations, suggesting a complex relationship between protein intake, the gut–oxidative-stress axis, and body composition.

## 1. Introduction

Malnutrition is a frequent and prognostically relevant complication of advanced chronic kidney disease (CKD), particularly among patients receiving maintenance hemodialysis. It results from inadequate energy and protein intake, the catabolic and inflammatory burden of kidney failure and dialysis, and dialysis-related nutrient losses [1,2]. Sarcopenia, characterized by loss of muscle mass and function, affects approximately 11% to 30% of patients with CKD, with the highest prevalence observed among those receiving dialysis, and is associated with increased mortality [2]. However, conventional measures such as body mass index (BMI) do not distinguish adipose tissue from lean tissue or extracellular fluid, an especially important limitation in patients receiving hemodialysis. Assessment of specific body-composition compartments may therefore provide more clinically and biologically relevant information than body size alone.

In patients receiving maintenance hemodialysis, protein-energy wasting and chronic systemic inflammation are closely and bidirectionally linked. Inflammation promotes anorexia and protein catabolism, whereas malnutrition may further increase susceptibility to inflammatory and vascular injury [3,4]. The gut–kidney axis may contribute to this interaction. Uremic dysbiosis and impaired intestinal barrier function promote the translocation of bacterial lipopolysaccharide (LPS) into the systemic circulation, resulting in endotoxemia, which is frequently observed in patients receiving hemodialysis [5,6]. Once in the circulation, LPS may promote skeletal muscle catabolism through Toll-like receptor 4 (TLR4)–nuclear factor kappa B signaling, with coordinated activation of the ubiquitin–proteasome and autophagy–lysosome proteolytic pathways [7].

Circulating LPS has also been associated with adiposity and adipose-tissue inflammation, raising the possibility that endotoxemia may show different relationships with fat and muscle compartments.

The LPS and protein-bound uremic toxins may also activate skeletal-muscle nicotinamide adenine dinucleotide phosphate (NADPH) oxidases, thereby promoting reactive oxygen species generation, mitochondrial dysfunction, and muscle proteolysis [8,9].

Oxidative stress is markedly increased in advanced CKD and may be further amplified by the hemodialysis procedure. NADPH oxidase 2 (NOX2) is an important enzymatic source of reactive oxygen species: it transfers electrons from NADPH to molecular oxygen, generating superoxide, which is subsequently converted to hydrogen peroxide (H_2_O_2_). Circulating soluble NOX2-derived peptide (sNox2-dp) provides an index of NOX2 activation, whereas H_2_O_2_ reflects a downstream component of oxidative stress [10,11]. Beyond the presence of abnormalities, the simultaneous assessment of endotoxemia and oxidative-stress markers may provide a more integrated view of the biological environment associated with body-composition alterations in hemodialysis. LPS can reflect gut-derived inflammatory exposure, whereas sNox2-dp and H_2_O_2_ capture complementary aspects of NOX2 activation and downstream reactive oxygen species generation. Evaluating these biomarkers together may help distinguish whether adipose- and muscle-related compartments are associated with different components of this pathway. Such an approach is particularly relevant in hemodialysis, where nutritional status, inflammation, oxidative stress, and fluid distribution are closely intertwined and may not be adequately represented by BMI alone.

Against this background, we specifically investigated the associations of circulating LPS, sNox2-dp, and H_2_O_2_ with bioimpedance-derived measures of adiposity and muscularity in patients receiving maintenance hemodialysis. Then, we examined whether endotoxemia and NOX2-related oxidative stress showed differential associations with fat and muscle-related body-composition compartments. We finally explored the relationships of normalized protein catabolic rate (nPCR), used as a surrogate of protein intake, with these biomarkers and body-composition measure.

## 2. Materials and Methods

### 2.1. Study Design

We conducted an observational, cross-sectional study at the Hemodialysis Unit of Fatebenefratelli Isola Tiberina Hospital in Rome, Italy, between March and December 2022. Adults aged ≥ 18 years receiving maintenance hemodialysis, who were enrolled in a clinical study protocol evaluating nutritional variables and body composition and were able to provide informed consent, were considered eligible for inclusion. Exclusion criteria were acute kidney injury, hemodynamic instability, clinically relevant fluid overload, or any acute or chronic highly catabolic condition likely to independently affect nutritional status, including active infection or sepsis. We also excluded patients with gastrointestinal symptoms at the time of enrollment, including vomiting, diarrhea, or constipation. Moreover, none of the participants were receiving antibiotics or probiotics during the study period or had received them in the period preceding enrollment. The study protocol was conducted in accordance with the Declaration of Helsinki and was approved by the local Ethics Committee (protocol no. 29/2013, approved on 28 June 2013). All participants provided written informed consent before enrollment.

### 2.2. Patient’s Characteristics

Demographic and clinical data, including age, sex, dialysis vintage, and major comorbidities, were collected from the patients’ medical records. Routine laboratory parameters, including serum creatinine, blood urea, electrolytes, fasting plasma glucose, albumin, lipid profile, and C-reactive protein, were obtained from blood samples collected before the scheduled hemodialysis session. Dialysis adequacy was assessed using Kt/V. All the patients used dialysis fistula as vascular access. Body mass index (BMI) was calculated as postdialysis body weight in kilograms divided by height in meters squared.

The normalized protein catabolic rate (nPCR), expressed as grams per kilogram per day, was estimated by urea kinetic modeling and normalized to postdialysis body weight. nPCR was used as a surrogate of habitual protein intake. Because nPCR may also be influenced by endogenous protein catabolism, it was interpreted as an indicator of protein intake in the context of clinical stability and in the absence of acute catabolic conditions [12].

### 2.3. Circulating Biomarkers of Endotoxemia and Oxidative Stress

Blood samples were collected from each participant before the second scheduled hemodialysis session of the week. Venous blood samples were collected in Vacutainer tubes with or without anticoagulant (3.8% Na citrate) and centrifuged at 1500× *g* for 10 min to obtain plasma or serum samples, respectively. Samples were then stored at −80 °C until analysis, as described below. Biomarker measurements were performed at the Laboratory of Platelet Pathophysiology, Department of Medical and Cardiovascular Science, Policlinico Umberto I, Sapienza University of Rome, Rome, Italy. Serum concentrations of soluble NOX2-derived peptide (sNox2-dp), a marker of NOX2 activation, were measured with a previously reported ELISA method [13]. sNox2-dp concentrations were determined using a reference curve generated with standards ranging from 0 to 200 pg/mL, as previously described [13]. Concentrations were expressed as pg/mL and intra- and inter-assay coefficients of variation (CVs) were 8.95% and 9.01%, respectively. Serum LPS concentrations were measured using a commercially available enzyme-linked immunosorbent assay kit (Cusabio, Houston, TX, USA; CSB-E09945h), according to the manufacturer’s instructions. The assay standards consisted of LPS purified from *Escherichia coli* J5. Optical density was measured at 450 nm, and LPS concentrations were expressed as pg/mL. The detection range was 6.25 pg/mL–400 pg/mL. Intra-assay and inter-assay coefficients of variation were <10%. Serum hydrogen peroxide (H_2_O_2_) concentrations were measured using a commercially available colorimetric assay (Abcam, Cambridge, UK; ab272537), according to the manufacturer’s instructions, and expressed as μM and intra- and inter-assay CVs were both <10%. The detection range was 0.2–30 μM.

### 2.4. Body-Composition Assessment by Bioimpedance Analysis

Body composition was assessed using a bioimpedance analyzer operating at 50 kHz (BIA 101, Akern, Florence, Italy). Measurements were performed by trained staff 1 h after completion of the second hemodialysis session of the week, with participants remaining in the supine position during the postdialysis equilibration period and throughout the examination, according to a previously described standardized protocol [14]. A standard tetrapolar electrode configuration was used, with current-injecting electrodes placed on the hand and foot and voltage-sensing electrodes placed on the wrist and ankle. Total body water (TBW) was estimated using the resistance extrapolated from the frequency; extracellular water (ECW), and intracellular water (ICW) were estimated using the manufacturer’s software. Fat-free mass (FFM) was calculated assuming a hydration fraction of 0.73 (FFM = TBW/0.73), and fat mass (FM) was calculated as the difference between postdialysis body weight and FFM. ICW was indexed to height squared (ICW/h^2^, L/m^2^) and used as a proxy of muscularity.

### 2.5. Statistical Analyses

Participant characteristics were summarized as mean ± standard deviation for approximately normally distributed continuous variables and as median with interquartile range for non-normally distributed continuous variables. The distribution of continuous variables was assessed using the Shapiro–Wilk test. Categorical variables were summarized as counts and percentages. Circulating biomarker concentrations were compared according to sex using the independent-samples Student *t* test or the Mann–Whitney U test, as appropriate according to their distribution, and their associations with age were examined using Spearman rank correlation. For analyses according to protein intake, participants were categorized into low- and high-nPCR groups using the median nPCR value of 0.89 g/kg/day as the cutoff. Biomarker concentrations were compared between the two nPCR groups using the independent-samples Student *t* test or the Mann–Whitney U test, as appropriate. Bivariate associations among circulating biomarkers, nPCR, and body-composition measures were assessed using Spearman rank correlation coefficients (rho). Correlation coefficients and corresponding *p* values were reported.

Two multivariable linear regression models were used to examine the independent associations of circulating biomarkers and nPCR with selected body-composition outcomes. In the first model, FM (kg) was the dependent variable, with LPS, nPCR, age, and sex entered as independent variables. In the second model, ICW/h^2^, L/m^2^, used as a proxy of muscularity, was the dependent variable, with sNox2-dp, nPCR, age, and sex entered as independent variables. Sex was entered as a binary variable, with male sex as the reference category. Unstandardized regression coefficients (β), 95% confidence intervals, standard errors, and *p* values were reported. All tests were two-sided, and *p* values < 0.05 were considered statistically significant. Analyses were performed using STATA, version [19.5] (StataCorp LLC, College Station, TX, USA).

## 3. Results

### 3.1. Clinical Characteristics of Hemodialysis Patients

Patient’s characteristics are summarized in Table 1. A total of 58 patients on chronic hemodialysis were enrolled, 37 of whom were male (64%). The mean age was 71 ± 14 years, and the median dialysis vintage was 46.6 months (20.5; 79.6). The mean body mass index (BMI) was 24.6 ± 4.2 kg/m^2^, indicating an overall normal-weight cohort. The most frequently observed comorbidity was arterial hypertension (91%), followed by diabetes mellitus (29%), ischemic heart disease (21%), peripheral artery disease (19%), and atrial fibrillation (7%) (Table 1).

Of the 58 patients, 56 underwent body-composition analysis; two patients were not evaluated (one had undergone lower-limb amputation due to diabetic vasculopathy, and one was transferred to another dialysis center).

Regarding muscularity indices, patients showed a mean ICW of 15.8 ± 4.3 L and an ICW/h^2^ of 5.8 ± 1.2 L/m^2^, with a mean ECW of 18.8 ± 3.7 L. The median FFM was 45.4 kg (39.1; 53.5), while the mean FM was 20.1 ± 7.9 kg (Table 1).

### 3.2. Oxidative Stress Biomarkers and Their Association with Muscularity Indices

No significant differences in the measured oxidative stress biomarkers were observed according to sex and no correlation was present with age.

The levels of LPS positively correlated with both sNox2-dp and H_2_O_2_ concentration (rho = 0.42, *p* = 0.001 and rho = 0.26, *p* = 0.05, respectively) (Table 2). Serum sNox2-dp levels also positively correlated with H_2_O_2_ concentration (rho = 0.352, *p* = 0.007) (Table 2).

The circulating levels of LPS and sNox2 positively correlated with BMI (rho = 0.410, *p* = 0.002; rho = 0.317, *p* = 0.018, respectively).

**Figure 1:**
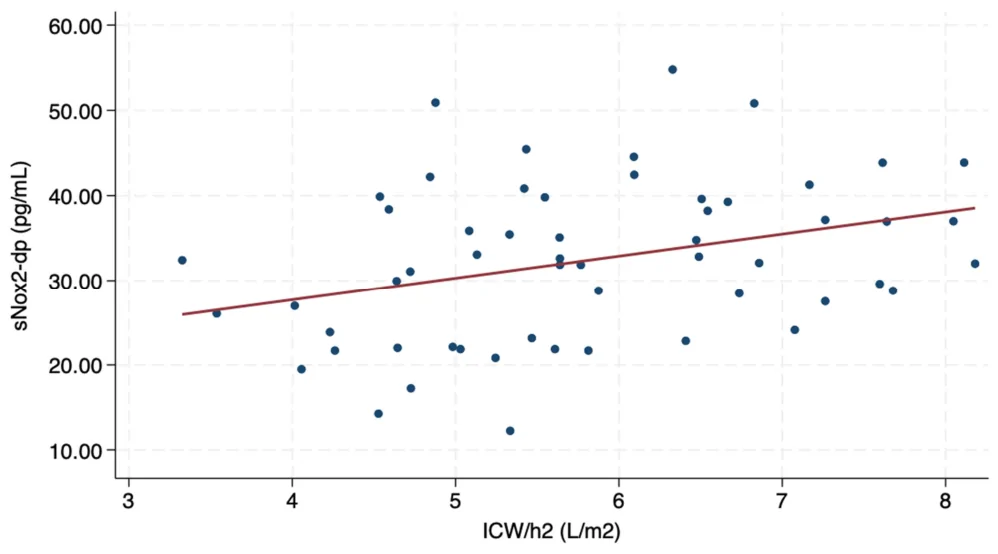
**Correlation between serum sNox2-dp and ICW/h^2^.** Correlation between serum sNox2-dp and intracellular water indexed to height squared (ICW/h^2^) in the study cohort. The solid line is the linear fit. sNox2-dp levels correlated positively and significantly with ICW/h^2^ (Spearman rho = 0.345, *p* = 0.010).

**Figure 2:**
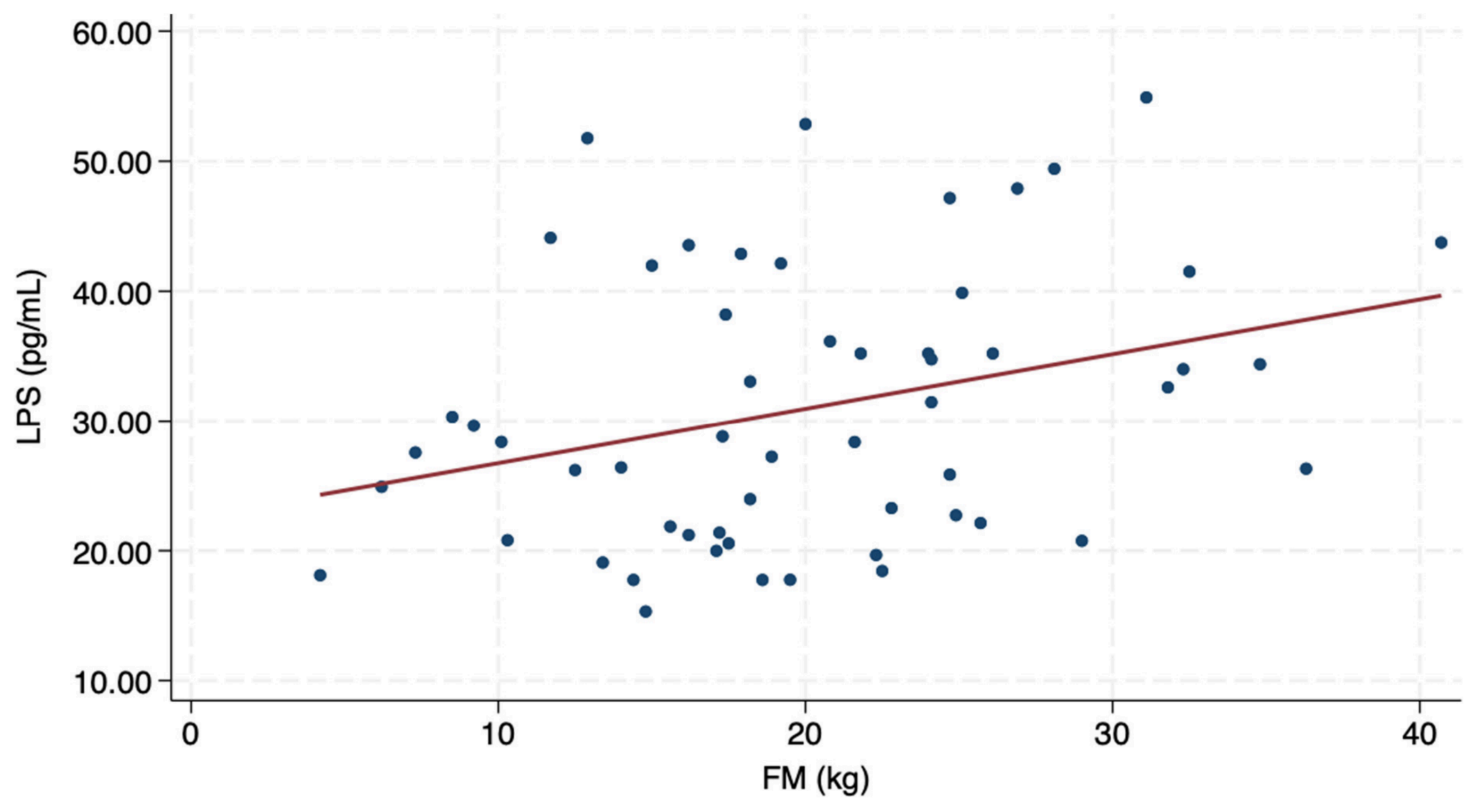
**Correlation between serum LPS and FM.** Correlation between serum lipopolysaccharide (LPS) and fat mass (FM) in the study cohort. The solid line is the linear fit. LPS levels correlated positively and significantly with FM (Spearman rho = 0.305, *p* = 0.023).

When considering body composition, sNox2-dp showed consistent positive correlations with markers of muscularity and intracellular hydration: FFM (rho = 0.382, *p* = 0.004), TBW (rho = 0.350, *p* = 0.008), ICW (rho = 0.346, *p* = 0.009), and ICW/h^2^ (rho = 0.345, *p* = 0.010) (Figure 1), whereas LPS positively correlated only with FM (rho = 0.305, *p* = 0.023, Figure 2) (see Table 3).

### 3.3. Association Between Oxidative Stress Biomarkers and nPCR

Based on median value of nPCR (grams per kilogram per day), we stratified patients into low (≤ 0.89) and high (>0.89) nPCR groups (n = 30 and n = 27, respectively).

**Figure 3:**
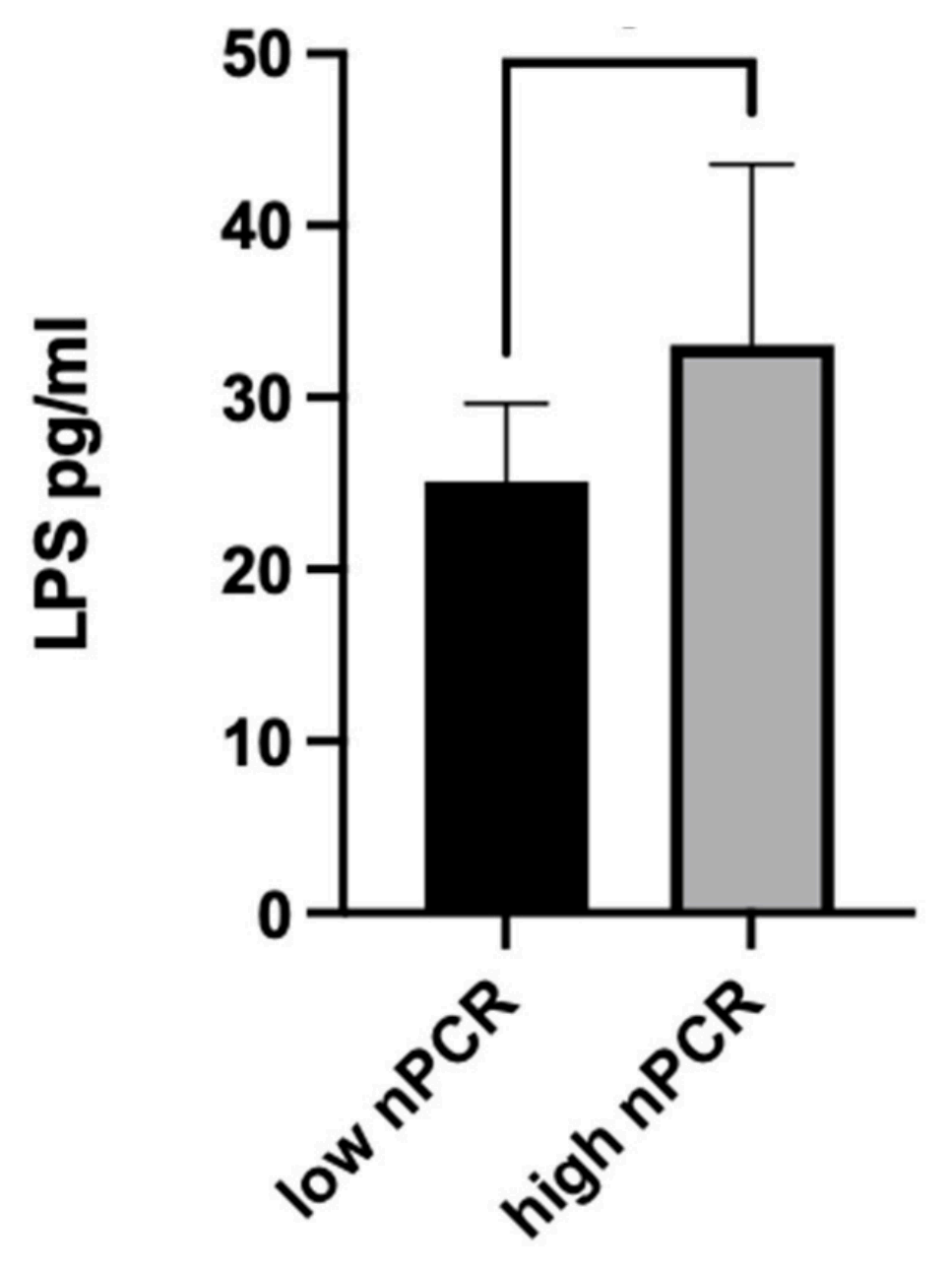
Circulating lipopolysaccharide (LPS) levels according to nPCR (below or over the median value). Bar graph showing serum LPS, pg/mL concentrations stratified by normalized protein catabolic rate (nPCR) into low-nPCR (n = 30) and high-nPCR (n = 27) groups by median split. Bars represent the 95% CI. LPS concentrations were significantly higher in the high-nPCR group than in the low-nPCR group (*p* = 0.015).

Lipopolysaccharide (LPS) concentrations were significantly higher in patients with high nPCR than in those with low nPCR (*p* = 0.015, Figure 3).

The H_2_O^2^ concentration showed a similar trend, tending to be greater in the high-nPCR group, although the difference did not reach statistical significance (*p* = 0.095), whereas sNox2-dp levels did not differ between the two groups (*p* = 0.79). nPCR positively correlated with ICW/h^2^ (rho = 0.36, *p* = 0.007), whereas no significant correlation was observed with fat mass (FM; rho = 0.03, *p* = 0.85).

### 3.4. Oxidative Stress and LPS: Multivariable Models for Body Composition

Two multivariable linear regression models were built to identify independent determinants of ICW/h^2^ and FM. In the first model, FM (kg) was regressed on LPS, nPCR, age, and sex (Table 4). Among the predictors, only LPS was independently associated with fat mass (β = 0.21, 95% CI 0.004 to 0.415, *p* = 0.046), whereas nPCR (β = 4.53, *p* = 0.41), age (β = 0.04, *p* = 0.63), and sex (β = 0.57 for female, *p* = 0.79) were not.

In the second model, ICW/h^2^ was regressed on sNox2-dp, nPCR, age, and sex (Table 5). Age was independently and inversely associated with ICW/h^2^ (β = −0.025, 95% CI −0.047 to −0.002, *p* = 0.034), nPCR showed a positive association (β = 1.39, 95% CI −0.001 to 2.79, *p* = 0.050), and sNox2-dp displayed a positive trend that did not reach statistical significance (β = 0.029, 95% CI −0.001 to 0.059, *p* = 0.060). Female sex was confirmed to be associated with muscularity (*p* = 0.008).

## 4. Discussion

In this cross-sectional study of prevalent chronic hemodialysis patients, we explored the associations between circulating markers of the gut–oxidative-stress axis—endotoxemia (LPS) and NADPH-oxidase activation (sNox2-dp) and its oxidative product (H_2_O_2_)—and body-composition parameters.

We observed that LPS, sNox2-dp, and H_2_O_2_ were mutually and positively correlated, supporting the existence of a coordinated axis. Also, sNox2-dp tracked consistently with indices of muscularity and intracellular hydration (FFM, TBW, ICW, and ICW/h^2^), whereas LPS tracked with adiposity (BMI and FM) and was the only independent determinant of fat mass in multivariable analysis.

The reciprocal correlations among LPS, sNox2-dp, and H_2_O_2_ are biologically coherent with the established pathway whereby gut-derived endotoxin activates innate immune cells through TLR4/CD14, driving assembly of NADPH oxidase (Nox2) and the generation of superoxide and H_2_O_2_ [7,15,16].

Endotoxemia is characteristically elevated as a consequence of gut-barrier disruption, circulatory stress, and reduced LPS-neutralizing capacity [15,17], and the dialysis procedure itself acutely increases reactive oxygen species production [11].

Notably, the circulating concentrations observed in our cohort were numerically higher than those previously reported in healthy individuals of comparable age [10]. This comparison supports the presence of a heightened endotoxemic and oxidative-stress milieu in maintenance hemodialysis, although differences in analytical methods across studies preclude direct quantitative comparisons.

Nevertheless, our findings are consistent with such an axis; the cross-sectional design may limit speculation on directionality or causality, and the observed variation may also reflect a shared inflammatory background rather than a strictly sequential pathway. Our observations suggest that endotoxemia and NOX2-related oxidative stress should not be interpreted as interchangeable markers of a single process. Their different relationships with fat mass and muscle-related compartments may reflect distinct biological sources, tissue responses, or metabolic contexts within the same inflammatory environment. This distinction may also help to explain why circulating oxidative-stress biomarkers do not uniformly parallel conventional body compartments in hemodialysis. At the same time, the observed associations cannot establish whether these biomarkers contribute to body-composition changes or instead reflect underlying differences in tissue mass, metabolic activity, or inflammation.

The preferential association of LPS with adiposity is supported by a substantial body of evidence.

In a cohort of long-term hemodialysis patients, high BMI was independently associated with higher endotoxemia [18]. This aligns with the broader concept of metabolic endotoxemia, in which LPS correlates with fat mass and promotes adipose-tissue dysfunction and inflammation [19,20,21], and with reports that inflammatory markers align with fat compartments rather than with lean mass in dialysis and ESRD patients [22]. In our analysis, the identification of LPS as independently associated with fat mass extends these observations to a directly measured body-composition phenotype.

The association of sNox2-dp with lean mass and intracellular-water compartments is the most novel of our observations and warrants careful interpretation, because part of the relevant literature links NADPH-oxidase activation to muscle loss rather than to muscle preservation. Advanced oxidation protein products induce muscle atrophy through a CD36/NADPH-oxidase/ROS pathway, with serum levels inversely related to muscle mass and grip strength [9], and F2-isoprostanes are independently and negatively associated with lean mass in CKD [23]. Our finding of a positive correlation between sNox2-dp and muscularity is not in contradiction with these data if the two roles of Nox2 are distinguished. Skeletal muscle constitutively expresses NOX2 and uses it for contraction- and insulin-stimulated redox signaling and metabolic adaptation [24]. A larger muscle compartment therefore represents a larger source of releasable sNox2-dp, independently of the pro-atrophic action of reactive oxygen species. Under this interpretation sNox2-dp behaves partly as a marker of muscle-derived enzyme burden rather than solely as a driver of wasting. Because FFM, TBW, and ICW are strongly influenced by sex and body size, we confirmed that these associations persisted after adjustment, reducing the likelihood that the association is explained by anthropometric measures confounding. The use of ICW as surrogate of muscle-cell mass is well founded. In fact, bioimpedance-derived intracellular water predominantly reflects muscle cell volume, the ECW/ICW ratio is a validated marker of muscle wasting and mortality in hemodialysis [25,26], and BIA is endorsed for body-composition assessment in maintenance hemodialysis by the KDOQI 2020 guideline [27].

Moreover, nPCR, often used as a surrogate of protein intake, was associated with both endotoxemia and muscularity. Patients with higher nPCR showed significantly greater circulating LPS concentrations, while nPCR positively correlated with ICW/h^2^ and remained positively associated with this marker at the threshold of statistical significance after adjustment for age, sex, and sNox2-dp. This finding is consistent with previous studies showing that higher nPCR or normalized protein nitrogen appearance is associated with greater lean body mass and skeletal muscle area in dialysis patients, whereas inadequate protein intake may contribute to protein-energy wasting [28,29].

At the same time, the association between higher nPCR and increased LPS raises the hypothesis that dietary protein exposure may be related to the gut–kidney axis. In CKD, greater protein delivery to the colon may potentially enhance proteolytic fermentation, promote dysbiosis, and impair intestinal barrier integrity, thereby facilitating the systemic translocation of bacterial products such as LPS [30,31,32]. These findings may therefore reflect a complex association whereby higher nPCR is linked to both greater muscularity and higher circulating LPS concentrations. Whether dietary protein intake contributes directly to endotoxemia cannot be ascertained from these data.

Our study has several limitations. The cross-sectional, single-center design and the relatively modest sample size preclude causal inference and limit statistical power, so that borderline associations (nPCR and sNox2-dp in the regression models) should be regarded as hypothesis-generating. In particular, although LPS was an independent predictor of fat mass, the overall fat-mass model explained only a small fraction of the variance, and this specific association therefore warrants confirmation in larger cohorts. We did not directly measure intestinal permeability, insulin resistance, or dietary composition, so the proposed metabolic-endotoxemia pathway remains inferential. The nPCR is a surrogate of protein rather than total caloric intake, and circulating LPS measurement is subject to known pre-analytical variability. Finally, residual confounding by comorbidity, dialysis vintage, and treatment-related factors cannot be excluded.

In conclusion, in chronic hemodialysis patients endotoxemia and NADPH-oxidase-derived oxidative stress showed distinct associations with body composition—LPS with adiposity and sNox2-dp with muscularity—within a potentially interconnected gut–oxidative-stress axis, with nPCR showing distinct associations with both muscle mass and circulating LPS. These findings suggest that gut-derived endotoxemia may be a relevant and potentially modifiable link between nutrition, inflammation, and adverse body-composition remodeling in this population, and merit confirmation in prospective studies.

## Figures and Tables

**Table 1:** Baseline characteristics of the study population.

Parameter	N = 58
Sex (M), n (%)	37 (64)
Age, years	73 (62; 82)
Dialysis vintage, months	46.6 (20.5; 79.6)
BMI, kg/m^2^	24.6 ± 4.2
nPCR, g/kg/d	0.89 (0.76; 0.98)
Kt/V	1.3 ± 0.3
Comorbidities	
Arterial hypertension, n (%)	53 (91)
Ischemic heart disease, n (%)	12 (21)
Peripheral artery disease, n (%)	11 (19)
Atrial fibrillation, n (%)	4 (7)
Diabetes mellitus, n (%)	17 (29)
Blood parameters	
Creatinine, mg/dL	8.6 (6.8; 11.2)
Blood urea, mg/dL	115.1 (85.9; 131.1)
Sodium, mEq/L	138.1 ± 2.4
Potassium, mEq/L	5.24 ± 0.94
Fasting plasma glucose, mg/dL	98 (87; 117)
Albumin, g/dL	3.8 (3.5; 4.0)
Total cholesterol, mg/dL	141 (113; 153.5)
LDL cholesterol, mg/dL	74.5 (56; 97.5)
Triglycerides, mg/dL	113.5 (80; 150.5)
C-reactive protein, mg/dL	0.35 (0.19; 0.74)
Body composition *	
ICW, L	15.8 ± 4.3
ICW/h^2^, L/m^2^	5.8 ± 1.2
ECW, L	18.8 ± 3.7
FFM, kg	45.4 (39.1; 53.5)
FM, kg	20.1 ± 7.9
Markers of inflammation and oxidative stress	
H_2_O_2_, μM	24.5 (16.1; 35.3)
LPS, pg/mL	28.4 (21.4; 38.2)
sNox2-dp, pg/mL	31.8 ± 9.9

Abbreviations: Body mass index, BMI; normalized protein catabolic rate, nPCR; low-density lipoprotein, LDL; C-reactive protein, CRP; intracellular water, ICW; extracellular water, ECW; fat-free mass, FFM; fat mass, FM; hydrogen peroxide, H_2_O_2_; lipopolysaccharide, LPS; soluble NADPH-oxidase 2-derived peptide, sNox2-dp. Normally distributed variables are expressed as mean ± SD; non-normally distributed variables as median (25th; 75th percentile); categorical variables as n (%). * N = 56.

**Table 2:** Correlations between serum biomarkers.

Variable Pair	rho	*p*-Value
LPS–Nox2-dp	0.4203	0.0011
sNox2-dp–H_2_O_2_	0.3524	0.0069
LPS–H_2_O_2_	0.2586	0.0502

Spearman rank correlation coefficient (rho); n = 58.

**Table 3:** Correlations of inflammatory/oxidative biomarkers with body-composition parameters.

	BMI	FFM	FM	TBW	ECW	ICW	ICW/h^2^
LPS	rho = 0.410 *p* = 0.002	rho = 0.186 *p* = 0.169	rho = 0.305 *p* = 0.023	rho = 0.155 *p* = 0.253	rho = 0.168 *p* = 0.215	rho = 0.170 *p* = 0.210	rho = 0.225 *p* = 0.096
sNox2-dp	rho = 0.317 *p* = 0.018	rho = 0.382 *p* = 0.004	rho = 0.219 *p* = 0.106	rho = 0.350 *p* = 0.008	rho = 0.201 *p* = 0.137	rho = 0.346 *p* = 0.009	rho = 0.345 *p* = 0.010
H _2 _O _2_	rho = 0.166 *p* = 0.221	rho = 0.010 *p* = 0.941	rho = 0.170 *p* = 0.210	rho = 0.028 *p* = 0.835	rho = 0.050 *p* = 0.716	rho = −0.007 *p* = 0.961	rho = 0.027 *p* = 0.844

Abbreviations: BMI, body mass index; FFM, fat-free mass; FM, fat mass; TBW, total body water; ECW, extracellular water; ICW, intracellular water. Spearman rank correlation coefficient (rho) and *p*-value; n = 56. Significant correlations (*p* < 0.05).

**Table 4:** Multivariable linear regression model for fat mass (FM, kg).

Predictor	β	95% CI	SE	t	*p*
LPS (pg/mL)	0.210	0.004 to 0.415	0.102	2.05	0.046
nPCR	4.527	−6.366 to 15.420	5.426	0.83	0.408
Age (years)	0.042	−0.133 to 0.217	0.087	0.48	0.631
Sex (female)	0.569	−3.738 to 4.876	2.146	0.27	0.792
Constant	6.415	−11.991 to 24.821	9.168	0.70	0.487

Dependent variable: fat mass (FM, kg). N = 56. β, unstandardized regression coefficient; CI, confidence interval; SE, standard error. Significant coefficients (*p* ≤ 0.05).

**Table 5:** Multivariable linear regression model for intracellular water normalized for height (ICW/h^2^).

Predictor	β	95% CI	SE	t	*p*
sNox2-dp (pg/mL)	0.029	−0.001 to 0.059	0.015	1.93	0.060
nPCR	1.393	−0.001 to 2.787	0.694	2.01	0.050
Age (years)	−0.025	−0.047 to −0.002	0.011	−2.18	0.034
Sex (female)	−0.804	−1.388 to −0.220	0.291	−2.76	0.008
Constant	5.678	3.140 to 8.216	1.264	4.49	<0.001

Dependent variable: intracellular water normalized for height (ICW/h^2^). N = 56. β, unstandardized regression coefficient; CI, confidence interval; SE, standard error. Significant coefficients (*p* ≤ 0.05).

## Data Availability

Due to privacy and ethical restrictions, the raw datasets generated, used, and analyzed in the current study are available from the corresponding author upon reasonable request.
